# Diagnostic accuracy of spirometry in primary care

**DOI:** 10.1186/1471-2466-9-31

**Published:** 2009-07-10

**Authors:** Antonius Schneider, Lena Gindner, Lisa Tilemann, Tjard Schermer, Geert-Jan Dinant, Franz Joachim Meyer, Joachim Szecsenyi

**Affiliations:** 1 Institute of General Practice, Technische Universität München, München, Germany; 2 Department of General Practice and Health Services Research, University Hospital, University of Heidelberg, Heidelberg, Germany; 3 Department of Primary Care Medicine, Radboud University Nijmegen Medical Centre, Nijmegen, The Netherlands; 4 Department of General Practice, School for Public Health and Primary Care, Maastricht University, Maastricht, The Netherlands; 5 Department of Cardiology, Pulmonology and Angiology, Medical Centre, University of Heidelberg, Heidelberg, Germany

## Abstract

**Background:**

To evaluate the sensitivity, specificity and predictive values of spirometry for the diagnosis of chronic obstructive pulmonary disease (COPD) and asthma in patients suspected of suffering from an obstructive airway disease (OAD) in primary care.

**Methods:**

Cross sectional diagnostic study of 219 adult patients attending 10 general practices for the first time with complaints suspicious for OAD. All patients underwent spirometry and structured medical histories were documented. All patients received whole-body plethysmography (WBP) in a lung function laboratory. The reference standard was the Tiffeneau ratio (FEV_1_/VC) received by the spirometric maneuver during examination with WBP. In the event of inconclusive results, bronchial provocation was performed to determine bronchial hyper-responsiveness (BHR). Asthma was defined as a PC_20 _fall after inhaling methacholine concentration ≤ 16 mg/ml.

**Results:**

90 (41.1%) patients suffered from asthma, 50 (22.8%) suffered from COPD, 79 (36.1%) had no OAD. The sensitivity for diagnosing airway obstruction in COPD was 92% (95%CI 80–97); specificity was 84% (95%CI 77–89). The positive predictive value (PPV) was 63% (95%CI 51–73); negative predictive value (NPV) was 97% (95%CI 93–99). The sensitivity for diagnosing airway obstruction in asthma was 29% (95%CI 21–39); specificity was 90% (95%CI 81–95). PPV was 77% (95%CI 60–88); NPV was 53% (95%CI 45–61).

**Conclusion:**

COPD can be estimated with high diagnostic accuracy using spirometry. It is also possible to rule in asthma with spirometry. However, asthma can not be ruled out only using spirometry. This diagnostic uncertainty leads to an overestimation of asthma presence. Patients with inconclusive spirometric results should be referred for nitric oxide (NO) – measurement and/or bronchial provocation if possible to guarantee accurate diagnosis.

## Background

Asthma is a common chronic disease with a high prevalence of approx. 5% in industrialized nations. It is characterized by a chronic inflammation process which induces bronchial hyper-responsiveness and in most cases, reversible airway obstruction [1]. Another common pulmonary disease is chronic obstructive pulmonary disease (COPD) which shows irreversible airway obstruction, and which is mostly caused by inhaling tobacco smoke [2]. The prevalence of COPD is estimated to be around 10% and expected to be the fourth most important cause of death in 2020 [3]. Due to this high morbidity, general practitioners play a key role in detecting the disease as they see patients during the earlier stages of disease. Spirometric investigation is seen as a gold standard for diagnosing airway obstruction. Therefore, office spirometry is increasingly seen as a quality standard in general practice [4,5].

The efficacy of spirometry in diagnosing COPD was demonstrated by a specialist team, which received referrals for performing spirometry and bronchodilator reversibility testing in patients suspected of having COPD [6]. The DIDASCO Study revealed the difficulty of diagnosing COPD with screening questionnaires only and concluded that spirometry is essential for early diagnosis [7]. These investigations focused on COPD only, which is marked by irreversible airway obstruction. The diagnostic value of spirometry for diagnosing asthma marked by reversible airway obstruction remains unclear. This is of importance, as asthma needs to be diagnosed by bronchial provocation testing when spirometry shows no airway obstruction [8]. One diagnostic study in primary care used spirometry and bronchial provocation testing for identifying patients with asthma and COPD [9]. However, this was only carried out in patients complaining of suffering from a cough; and spirometry was performed by a single specialist. Spirometry and bronchial provocation testing were also used in the DIMCA study [10]. Indeed this was a screening study performed in a specialist center to detect patients in early stadiums of asthma or COPD.

Due to the design of these asthma and COPD trials, there is no evidence of the diagnostic accuracy of spirometry itself. Therefore, the true degree of the associated diagnostic uncertainty for patients with complaints suspected of having an airway obstruction remains unclear. The need for closing this gap of knowledge has been pointed out several times [11,12]. The difficulty is that the pretest probability of a disease and its severity in primary care is lower when compared to a hospital setting, thus hampering the predictive values of diagnostic tests [13,14]. Therefore, test results evaluated in hospital settings can not easily be transferred into general practice [15]. The aim of this study was to investigate the sensitivity, specificity and predictive values of spirometry for diagnosing airway obstruction in asthma and COPD in general practice.

## Methods

### Design and sample

This cross-sectional study was performed between January 2006 and December 2007 with fourteen general practitioners (GPs) working in ten general practices. 219 patients visiting their GP for the first time with complaints suggestive of obstructive airway disease (OAD) were consecutively included in each practice. Patients visited their GPs with symptoms such as dyspnea, coughing or expectoration. Their medical history was taken with a structured questionnaire. The patients had not been diagnosed previously for OAD and they had not received any previous anti-obstructive medicine. Other exclusion criteria related to well known contra-indications for bronchodilator reversibility testing or bronchial provocation, namely untreated hyperthyreosis, unstable coronary artery disease, and cardiac arrhythmia. Pregnancy also led to exclusion. The study was approved by the Medical Ethics Committee of the University of Heidelberg. Patients gave written informed consent.

On the basis of the pilot study [16] we estimated the pre-test probability of asthma as being 45% and of COPD as 16%. We estimated the sensitivity for diagnosing asthma to be 30% and the specificity to be 90%. The sensitivity and specificity for finding COPD was each estimated to be 90%. Power calculations showed that we had to include at least 208 patients to determine the sensitivities and specificities with a 95% confidence interval of ± 10% [17]. The diagnostic values of spirometry in general practice were calculated separately for each asthma and COPD group to avoid confusion. The diagnostic value for diagnosing asthma under optimal conditions was investigated by pooling all patients and determining the sensitivity, specificity and predictive values of spirometric maneuvers of the lung function laboratory.

### Index test: Spirometry in general practice

Ten general practices were equipped with the same electronic spirometer (Medikro SpiroStar USB^®^) and associated spirometry software. The spirometer was a hand-held instrument for lung function testing that has to be connected via USB device to a computer. Spirometric data, flow-volume and volume-time graphs are displayed in real-time on the personal computer as the patient performs the spirometry test. A calibration file saves the calibration data for internal quality assurance. Instrument performance is regularly monitored and performance deviations are identified by the software. The software also compares the measured values with reference tables. The best of three consecutive spirometry recordings was used in accordance with the guidelines of the European Respiratory Society [18]. The maximal inspiratory and expiratory flow volume curves were generated by forced deep inspiration and expiration with short intervening periods of tidal breathing; patients used a nose clip. The maneuver was performed in a sitting position. Patients with a FEV_1 _(forced expiratory volume in one second) < 80% of predicted received a bronchodilation test with an additional performance of spirometry 20 minutes after inhaling salbutamol. Obstructive airway disease was diagnosed if FEV_1_/VC ≤ 70% and/or FEV_1 _< 80% [4,5]. Obstruction was considered to be reversible on salbutamol (which indicates a diagnosis of asthma) if the bronchodilation response Δ FEV_1 _was ≥12% of the baseline and ≥200 ml [4] and norm values were reached. GPs were asked to make their diagnoses based on the test results.

All of the GPs were appropriately trained in the key aspects of the diagnosis and management of asthma and COPD, as well as in performing and interpreting spirometry during two educational meetings. The practice assistants completed an intensive 6-hour course and were trained in performing and interpreting spirometry. Two outreach visits were also performed with repeated individual education at a direct, practical level, until the optimal performance of the spirometry was secured.

### Reference test: Bodyplethysmography and bronchial provocation

After diagnosis by their GP, all patients were referred to the lung function laboratory of the University Medical Hospital at once for investigation with whole-body plethysmography (WBP). If therapy was necessary due to asthma or COPD, it was initiated by the GP. However, patients were instructed not to use any bronchodilator or inhaled steroid twelve hours before visiting the lung function laboratory. Spirometry is normally the routine method for measuring the lung volume required to diagnose airflow obstruction – i.e. (forced) vital capacity ((F)VC) or FEV_1_. However, spirometry is not capable of providing information about intrathoracic residual volume or total airway resistance. A WBP is required to measure residual volume (RV), functional residual capacity (FRC), and total lung capacity (TLC). Therefore, the advantage of WBP over spirometry is that it is able to distinguish between restrictive and obstructive processes. Additionally, the resistance to airflow can be evaluated and the response of airway resistance, airway conductance and thoracic gas volume can be determined in response to bronchodilator reversibility testing and bronchial provocation. In particular circumstances, measurement of these lung volumes are strictly necessary for a correct physiological diagnosis [19,20]. However, as WBP is only common in highly developed health care systems and the added value on top of spirometry remains unclear, it is only recommended in a few guidelines [21-23].

#### Measurement technique of whole-body plethysmography and bronchial provocation

**Figure 1:**
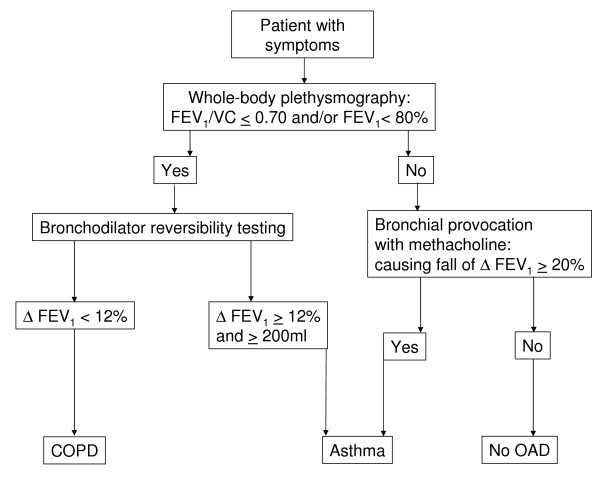
**Diagnostic decision making with the reference standard (whole-body plethysmography and bronchial provocation) in the lung function laboratory (COPD = Chronic obstructive pulmonary disease; OAD = obstructive airway disease)**.

During WBP, the patient sits inside an airtight chamber and makes respiratory efforts against the closed shutter, causing chest volume to expand and decompressing the air in the lungs. The increase in chest volume reduces the box's volume, thus increasing the pressure in the box. The procedures were performed according to standard protocols [21]. Lung function reference values that had been adjusted for sex, age, and height were used [24]. Patients with FEV_1 _< 80% of predicted received a bronchodilation test with an additional performance of WBP 20 minutes after inhaling salbutamol. An obstructive airway disease was diagnosed if FEV_1 _< 80% and/or FEV_1_/VC ≤ 0.70. The obstruction was classified as reversible on a salbutamol (indicating asthma) when Δ FEV_1 _was ≥12% and ≥200 ml from the baseline value [4] and norm values were reached. In all other cases, the obstruction was classified as not reversible (Figure 1). If there was no bronchial obstruction, bronchial provocation was performed to determine bronchial hyper-responsiveness (BHR). Bronchial provocation is considered to be the best method for diagnosing asthma [25], although there is conflicting evidence [26], probably arising from variations in the population studied, as the diagnostic value increases with pre-test probability of the disease [27]. Trained lung function technicians measured bronchial hyper-responsiveness to methacholine according to the ATS guideline [8]. A diagnosis of 'asthma' was made if there was a 20% fall in FEV1 (PC_20_) from the baseline value after inhaling methacholine stepwise until the maximum concentration (16 mg/ml). The pneumologist was blinded against the diagnosis of the GP.

### Data analysis

**Figure 2:**
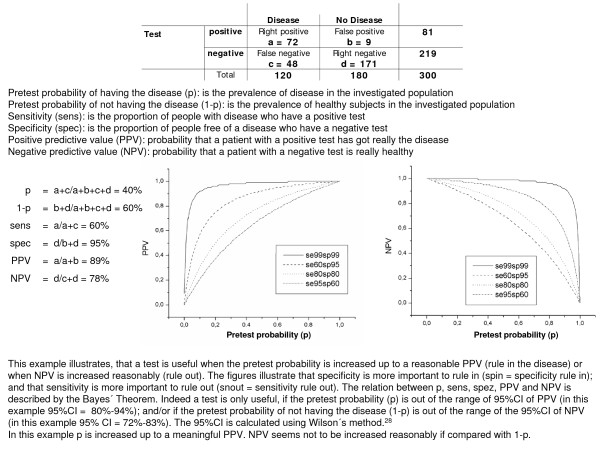
**Calculation example for the relation between pretest probability, sensitivitiy, specificity, positive predictive value (PPV) and negative predictive value (NPV)**.

The baseline data were presented descriptively. The sensitivity, specificity, and predictive values of the spirometric investigation (FEV_1 _and/or FEV_1_/VC) in general practice were calculated with two-by-two contingency tables with the diagnosis of the pneumologist (WBP and bronchial provocation) as 'gold standard'. The data were analyzed with SPSS 15.0 for Windows. 95% confidence intervals were calculated using Wilson's method [28] with the statistical package CIA (Confidence Interval Analysis) [29]. An explanation of how to interpret PPV and NPV is provided in figure 2.

## Results

### Study population

**Figure 3:**
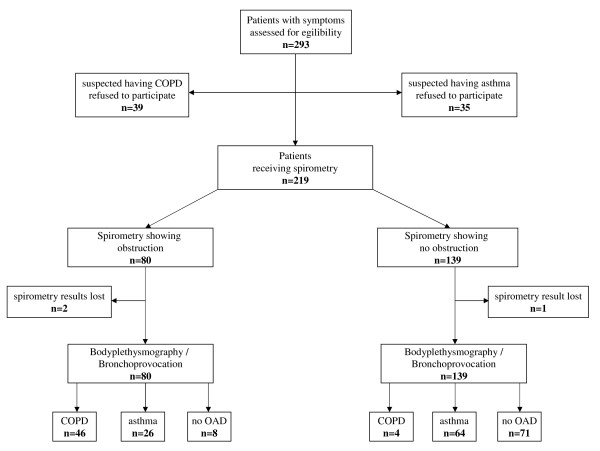
**Flow chart of inclusion and diagnostic work up (COPD = Chronic obstructive pulmonary disease; OAD = obstructive airway disease; OAD = obstructive airway disease)**.

A total of 293 patients were assessed for eligibility (Figure 3). 74 patients received spirometry but did not want to receive whole body plethysmography and eventually bronchial provocation. Therefore, altogether 219 patients participated in the study (127 [57.7%] were female) (Table 1). The average age was 43.8 years. The average body mass index (BMI) was 25.3 (SD 4.4). Of the participating patients, 78 (35.6%) showed airway obstruction in general practice and 138 (63.0%) no abnormal findings in spirometry (Figure 3). Three spirometric results were lost to follow-up. According to the diagnostic decision making in the lung function laboratory, 90 (41.1%) patients had asthma, 50 (22.8%) of the participating patients had irreversible airway obstruction (COPD), and 79 (36.1%) showed no abnormal findings. A diagnosis of asthma was made in 76 of the cases with bronchial provocation, with only 14 patients identified solely on the basis of bronchodilator reversibility testing. The decision that the bronchial provocation was positive was made in 74 cases by 20% fall of FEV_1 _and in two cases by extreme increase of airway resistance accompanied by development of clinical symptoms of asthma during bronchial provocation. There were no significant differences in sex (p = 0.719) or obesity (BMI ≥ 30) (p = 0.272) between the diagnoses (chi-square test).

**Table 1:** Characteristics of the study population. Values are number (proportion) or mean (SD); OAD = obstructive airway disease; COPD = Chronic obstructive airway disease (n = 219)

	**Overall** **n (%)**	**Asthma** **n (%)**	**COPD** **n (%)**	**No OAD** **n (%)**
n	219 (100)	90 (100)	50 (100)	79 (100)
Female	127 (57.7)	55 (61.1)	26 (54.1)	46 (58.2)
Obesity	30 (13.7)	8 (8.9)	10 (20.0)	12 (15.2)
Age (mean in years [sd])	43.8 [15.6]	37.9 [14.4]	56.9 [11.5]	42.1 [14.4]
Do you sometimes suffer from shortness of breath? (yes)	135 (61.4)	55 (61.1)	39 (76.3)	41 (51.9)
Have you suffered from wheezing in your chest? (yes)	108 (49.1)	47 (52.2)	30 (63.2)	30 (38.0)
Do you often suffer from a cough? (yes)	126 (57.3)	39 (43.3)	32 (65.8)	55 (69.6)
Do you often suffer from expectoration? (yes)	74 (33.6)	22 (24.4)	20 (36.8)	32 (40.5)
Have you been woken up with a feeling of tightness in your chest? (yes)	49 (22.3)	27 (30.0)	9 (10.5)	13 (16.5)
Have you been woken up by an attack of shortness of breath? (yes)	48 (21.8)	24 (26.7)	10 (18.4)	14 (17.7)
Have you suffered an asthma attack? (yes)	14 (6.4)	11 (12.2)	2 (2.6)	1 (1.3)
Do you suffer from any nasal allergies? (yes)	92 (41.8)	44 (48.9)	14 (31.6)	34 (43.0)
Do you often suffer from a common cold? (yes)	73 (33.2)	18 (20.0)	18 (26.3)	37 (46.8)
Do you smoke or did you smoke? (yes)	118 (53.4)	35 (38.9)	43 (86.8)	39 (49.4)
How much do/did you smoke? (mean in pack year [SD])	11.6 [17.7]	6.6 [12.9]	28.8 [21.7]	6.4 [11.9]

### Performance of spirometry in general practice

Spirometry was performed with full adherence to ERS guidelines in 86 (39.8%) cases (Table 2). There was moderate adherence to ERS in 82 (38.0%) cases. In 48 (22.2%) cases the ERS criteria were not fulfilled. E.g., the flow-volume curves were deformed or not exactly reproduced. Altogether, 78 (36.1%) spirometric maneuvers showed airway obstruction. However, a bronchial reversibility test was only performed in 37 (47.4%) of these 78 cases.

**Table 2:** Performance of spirometry in general practice (n = 216)

**Interpretation of flow-volume curve**	**n (%)**	**Bronchodilation test**	**n (%)**
Full adherence to ERS	86 (39.8)	Was not necessary	138 (36.1)
Adherence to ERS but only two flow-volume curves	69 (31.9)	Was necessary and performed	37 (17.1)
No adherence to ERS but first flow-volume curve perfect and showing no pathological signs	13 (6.0)	Was necessary but not performed	41 (19.0)
No adherence to ERS showing no obstruction	15 (6.9)		
No adherence to ERS indicating airway obstruction	33 (15.2)		

### Estimates of diagnostic accuracy of spirometry in general practice

In relation to the COPD diagnosis, 26 patients were diagnosed false positive (Table 3). 12 of these spirometric maneuvers showed full/moderate adherence, and 14 were not according to guidelines. Four patients were diagnosed as false negative as the forced maneuvers in spirometry were performed weakly, thus resulting in a virtually normal Tiffeneau ratio. In these cases was FEV_1 _> 80% of predicted and FEV1/VC < 0.70 in the WBP as reference standard. Sensitivity was 92% and specificity 84%. Thus the pretest probability could be enhanced reasonably from 23% to a posttest probability (PPV) of 63%; and COPD could be ruled out with high certainty (NPV 97%).

**Table 3:** 2 × 2 table of spirometry for diagnosing airway obstruction in patients with COPD (n = 208; asthma patients with FEV_1 _<80% of predicted in general practice and in lung function laboratory excluded)

	COPD	No COPD	
Spirometry +	44	26	
Spirometry -	4	134	
			208
Pretest probability of having COPD 23%			
Pretest probability of not having COPD 77%			
Sensitivity	92% (95%CI 80–97)		
Specificity	84% (95%CI 77–89)		
PPV	63% (95%CI 51–73)		
NPV	97% (95%CI 93–99)		

63 patients with asthma were diagnosed false negative as they showed no abnormal findings in spirometry (Table 4). It was only possible to identify them through bronchial provocation. Eight patients were diagnosed false positive; two of these spirometric maneuvers showed good adherence, and six were not according to guidelines. The pretest probability was enhanced from 41% up to 77%. However, asthma could not be ruled out, since NPV (53%) was similar to the pretest probability of 'not having asthma' (1-p = 59%); and 1-p was within the confidence interval of NPV (95%CI 45–61). The spirometric results as a part of the WBP investigation in the lung function laboratory are given in Table 5. Only 14 patients were identified by airway obstruction FEV_1 _< 80% of predicted and positive bronchial reversibility testing. In addition to this, under these optimal conditions with optimal differentiation between asthma and COPD, the sensitivity for diagnosing asthma solely on basis of spirometric maneuvers was only 16%. Again, NPV was similar to the pretest probability of 'not having asthma'.

**Table 4:** 2 × 2 table of spirometry for diagnosing airway obstruction in patients with asthma in general practice (n = 168; patients with COPD excluded)

	asthma	no asthma	
Spirometry +	26	8	
Spirometry -	63	71	
			168
Pretest probability of having asthma 41%			
Pretest probability of not having asthma 59%			
Sensitivity	29% (95%CI 21–39)		
Specificity	90% (95%CI 81–95)		
PPV	77% (95%CI 60–88)		
NPV	53% (95%CI 45–61)		

**Table 5:** 2 × 2 table of spirometry for diagnosing airway obstruction in patients with asthma in lung function laboratory (all patients included with differentiation between asthma and COPD)

	asthma	no asthma	
Spirometry +	14	0	
Spirometry -	76	129	
			219
Pretest probability of having asthma 41%			
Pretest probability of not having asthma 59%			
Sensitivity	16% (95%CI 10–24)		
Specificity	100% (95%CI 97–100)		
PPV	100% (95%CI 79–100)		
NPV	63% (95%CI 56–69)		

### Diagnostic decision making by the GPs

The comparison of the diagnoses by the general practitioners with the diagnoses of the pneumologists demonstrated a reasonable agreement with respect to COPD (Table 6). Additionally, the GPs suspected asthma correctly in 76.7% of asthma cases despite the diagnostic uncertainty using spirometry. Indeed the prevalence of asthma was overestimated with 58.2% of healthy subjects suspected of having asthma; and 7.8% of patients with asthma were considered to be healthy.

**Table 6:** Agreement between pneumologists' and general practitioners' diagnoses

**Pneumlogist\GP**	**Asthma**	**COPD**	**No OAD**	**Restrictive lung disease**
	**n (%)**	**n (%)**	**n (%)**	**n (%)**
Asthma (n = 90)	69 (76.7)	14 (15.5)	7 (7.8)	0 (0)
COPD (n = 50)	7 (16.2)	41 (82.0)	1 (2.7)	1 (0)
No OAD (n = 79)	46 (58.2)	8 (10.1)	25 (31.6)	0 (0)

## Discussion

To our knowledge, this is the first study evaluating the diagnostic accuracy of spirometry for diagnosing airflow obstruction in patients with asthma or COPD in primary care. We found that the use of spirometry is feasible within general practice after training GPs and practice nurses. Under these conditions, the presence or absence of COPD can be estimated with a comparatively high diagnostic accuracy. It is also possible to rule in asthma. However, it was impossible to rule out asthma as the sensitivity was too low.

The prevalence of COPD is increasing in nearly all countries of the world and a high diagnostic accuracy is a prerequisite of optimal therapeutic management. The important role of spirometry for diagnosing airway obstruction has already been demonstrated [6,7,30,31]. However, the diagnostic accuracy of spirometry for diagnosing COPD has been unknown up to now, thus leading to diagnostic uncertainty in suspected cases of COPD. Our results demonstrate that the pretest-probability of 22% of patients presenting themselves with complaints suggestive of airway obstruction can be increased up to a post-test probability of 63% for having COPD. This comparatively low PPV might be surprising, as the sensitivity was 84% and specificity was 92%. However, this is explainable by the low pretest probability. Another reason might be due to sub-maximal maneuvers, leading to false positive results by underestimation of FEV_1 _[11]. As a consequence, more efforts in terms of continuous education would be necessary for an improvement of performance and an interpretation of spirometry. Nevertheless, COPD can be definitively excluded (NPV 97%) when spirometry is performed optimally. For these reasons, spirometry should be used regularly for diagnosing and managing COPD in primary care.

In contrast to these promising results is the limited value of spirometry in excluding asthma. This might be explained by the reversibility of airway obstruction in asthma. It proved possible to speculate that patients with mild or moderate asthma show no airway obstruction when spirometry is performed. In these cases, it was necessary for the GP to estimate the presence or absence of asthma on the basis of the patient history and inconclusive spirometry. This was misleading in 53 (24.2%) of cases (46 patients false positive and 7 patients false negative). Therefore, alternative methods need to be found for diagnosing asthma in primary care. Guidelines recommend using the measurement of peak-flow-variability to diagnose asthma in case of inconclusive spirometry. However, the low diagnostic value of peak-flow-variability in primary care has already been demonstrated [32]. The SAPALDIA study, which used an epidemiologic approach, has also shown a poor diagnostic value [33]. The measurement of exhaled nitric oxide (NO) which is elevated in eosinophilic airway inflammation [34] has been shown to be more promising [35], although the technology is expensive. Therefore, patients suspected of having asthma might be tested with NO measurement or should be referred for bronchial provocation if possible to guarantee accurate diagnosis. Nevertheless, spirometry should be used in diagnosing asthma, as the positive predictive value has been comparatively high in general practice.

One important limitation was that 22% of the spirometric maneuvers were not performed correctly in general practice. However, with the analysis of the spirometric maneuvers as part of the WBP investigation in the lung function laboratory, we received accurate diagnostic values of spirometry. Our results revealed that the predictive values of general practice were slightly lower than in the lung function laboratory. In addition to this, it was not possible to include all patients consecutively, as some patients were not willing to travel to the lung function laboratory of the Medical Hospital. This might have led to an overestimation of the diagnostic accuracy of spirometry [36]. However, that would also emphasize the impossibility of excluding asthma solely with spirometry. Another limitation is due to the choice of the cut-off points. Our use of the ratio FEV_1_/VC ≤ 0.70 as is still recommended by GOLD [5] may have led to some overestimation of airway obstruction in older patients [37] and underestimation in younger patients [38]. The ATS/ERS guideline therefore suggests using lower limits of normal, which is statistically defined by the 5^th ^lower percentile of a reference population, to provide more accurate diagnoses [19]. This diagnostic algorithm was not integrated in the spirometric software at the time of our study. Moreover, we are aware of the limitations of a one-off lung function test to determine a final diagnosis, as a negative bronchodilator response can occur due to fixed airway obstruction in asthma. A trial of steroids might have been necessary to differentiate between asthma and COPD in some patients. Nevertheless, these limitations do not hamper our finding that asthma cannot be excluded solely with spirometry. The WBP showed little added value on top of spirometry. We used it as a reference standard to distinguish between overlapping diseases, COPD and restrictive lung disorder. However, we only experienced two changes in making the diagnosis with the added information of WBP. In two patients suffering from dyspnea attacks, the airway resistance was very high during bronchial provocation, but FEV_1 _remained normal. Moreover, we found no patient with restrictive lung disorder, which indicates a low prevalence in primary care settings. Therefore, the added value of WBP for primary care is limited and it should be reserved for patients who are difficult to diagnose and show persistent complaints.

It was not possible to specify the alternate diagnosis of the patients with no OAD, which is a typical problem of diagnostic studies in primary care. It was impossible to perform every investigation (e.g. gastroscopy to determine gastro-oesophageal reflux; x-ray) until a definite diagnosis could be made. This would not have been allowed by the Ethics Committee. However, this limitation does not alter the results of spirometric investigation. Finally, the participating GPs and practice assistants were highly motivated and received intensive training. Nevertheless, 22% of the spirometric maneuvers showed no guideline adherence. In particular bronchodilation testing was not performed regularly which might be due to organisational reasons and time constraints in general practice. The GPs estimated fourteen patients to suffer from COPD. However, the final pneumologists' diagnosis of these patients was asthma due to positive bronchodilator testing. Therefore, this lack of performance led the GPs to over-estimate COPD and under-estimate asthma in patients with airway obstruction. This is of importance as patients with asthma need to be treated preferably with inhaled steroids. However, our results are better than demonstrated by Miravitlles et al. [39], which might be due to the repeated education of the whole practice team. Nevertheless, these results are not satisfying enough. Further efforts are necessary to improve the performance of spirometry, as this could enhance the diagnostic accuracy. It has already been established that GPs are able to perform and interpret spirometry after educational meetings [40] and that performing spirometry has a positive impact on medical decision making [6,30,41]. It therefore seems reasonable and valuable to implement high quality spirometry in primary care.

## Conclusion

COPD can be estimated with high diagnostic accuracy using spirometry. It is also possible to rule in asthma with spirometry. However, asthma can not be ruled out only using spirometry. This diagnostic uncertainty leads to an overestimation of asthma presence. Patients with inconclusive spirometric results should be referred for NO – measurement and/or bronchial provocation if possible to guarantee accurate diagnosis.

## Competing interests

The authors declare that they have no competing interests.

## Authors' contributions

AS designed the study, performed the analyses and wrote the manuscript. LG trained the practice assistants, managed the data and helped to write the manuscript. LT helped to manage the data and to write the manuscript. TS and GJ helped to interpret the data and with writing. FJM made the final diagnoses as pneumologist and helped to write the manuscript. JS helped to write the manuscript. All authors read and approved the final manuscript.

## Pre-publication history

The pre-publication history for this paper can be accessed here:


